# Relationship of Iron Intake, Ferritin, and Hepcidin with the Transverse Relaxation Rate of Water Protons in the Pancreas

**DOI:** 10.3390/nu15173727

**Published:** 2023-08-25

**Authors:** Wandia Kimita, Juyeon Ko, Maxim S. Petrov

**Affiliations:** School of Medicine, University of Auckland, Auckland 1142, New Zealand

**Keywords:** iron metabolism, pancreas, pancreatitis, water protons, magnetic resonance imaging

## Abstract

(1) Background: There is a paucity of markers of iron metabolism in health and disease. The aim was to investigate the associations of iron metabolism with pancreas transverse water proton relaxation rate (R2_water_) in healthy individuals and people after an attack of pancreatitis. (2) Methods: All participants underwent a 3.0 T magnetic resonance imaging of the abdomen on the same scanner. High-speed T2-corrected multi-echo (HISTO) acquisition at single-voxel magnetic resonance spectroscopy and inline processing were used to quantify pancreas R2_water_. Habitual dietary intake of iron was determined using the EPIC-Norfolk food frequency questionnaire. Circulating levels of ferritin and hepcidin were measured. Generalised additive models were used, adjusting for age, sex, body mass index, and haemoglobin A1c. (3) Results: A total of 139 individuals (47 healthy individuals, 54 individuals after acute pancreatitis, and 38 individuals after chronic pancreatitis) were included. Total dietary intake of iron was significantly associated with pancreas R2_water_, consistently in healthy individuals (*p* < 0.001), individuals after acute pancreatitis (*p* < 0.001), and individuals after chronic pancreatitis (*p* < 0.001) across all the statistical models. Ferritin was significantly associated with pancreas R2_water_, consistently in healthy individuals (*p* < 0.001), individuals after acute pancreatitis (*p* < 0.001), and individuals after chronic pancreatitis (*p* = 0.01) across all adjusted models. Hepcidin was significantly associated with pancreas R2_water_ in individuals after acute pancreatitis (*p* < 0.001) and individuals after chronic pancreatitis (*p* = 0.04) in the most adjusted model. (4) Conclusions: Pancreas R2_water_, corrected for T2, is related to iron metabolism in both health and pancreatitis. This non-invasive marker could be used for automated in vivo identification of intra-pancreatic iron deposition.

## 1. Introduction

The global incidence of pancreatitis is high (33.7 cases for acute pancreatitis and 9.6 cases for chronic pancreatitis per 100,000 person-years) in the general population and is projected to increase notably by 2050 [1]. Mounting evidence shows that individuals after an attack of pancreatitis often have oxidative stress and develop a wide array of metabolic sequelae (e.g., new-onset diabetes, exocrine pancreatic dysfunction, osteoporosis) long after the clinical resolution of pancreatitis [2,3,4,5,6]. Further, 10% and 36% of individuals with the index attack of pancreatitis and recurrent pancreatitis, respectively, develop chronic pancreatitis, making early detection of pancreatitis progression and the development of sequelae critical in the management of people after an attack of pancreatitis [3]. Incremental advances in magnetic resonance (MR) techniques over the past decades have solidified the role of MR imaging as a key diagnostic and prognostic tool in patients with pancreatitis. Specifically, MR cholangiopancreatography has been useful in identifying pancreatic duct abnormalities and detection of pancreatic duct stones [7,8,9], T1 mapping—in the determination of early fibrosis of the pancreas [10,11], and proton density fat fraction derived from chemical-shift encoded MR imaging—in the quantification of intra-pancreatic fat deposition [12,13].

Relaxation rate R2 is an MR imaging parameter that could be complementarily employed in individuals with pancreatitis as changes in relaxivity could be associated with altered pancreas water content (resulting from oedema or synthesis of collagen fibres, in which protons are less abundant and tightly bound). The measurement of the relaxation rate is based on the physical aspects of nuclei relaxation to the ground state that occurs after exchangeable protons bound to iron-containing proteins and bulk water engage in a proton chemical exchange [14,15]. Further, the ability of MR to separate the water resonance from other confounding proton resonance sources (e.g., lipids) enables the direct analyses of the transverse relaxation rate of tissue water (R2_water_) [16,17]. The high-speed T2-corrected multi-echo (HISTO) H^1^ MR spectroscopy technique, where a single voxel sequence allows for quantification of tissue R2_water_, has been developed [18,19]. Relaxometry has been widely employed in measuring myocardial and hepatic siderosis [20,21], and liver R2_water_ has been studied in relation to hepatic iron content [18,22]. However, the relevance of pancreas R2_water_ to iron metabolism has been investigated in neither health nor pancreatitis.

The primary aim of this study was to investigate the associations of pancreas R2_water_ and iron metabolism (assessed holistically based on both habitual dietary iron intake and circulating markers of iron metabolism such as ferritin and hepcidin) in healthy individuals as well as those after acute pancreatitis and chronic pancreatitis. The secondary aim was to investigate the associations of liver R2_water_ with iron metabolism in the same individuals.

## 2. Materials and Methods

### 2.1. Study Design and Population

This was a cross-sectional study of healthy adults (≥18 years) and adults with a history of pancreatitis as part of the ARIES project [23]. Healthy individuals did not have a personal or family history of pancreatic diseases, diabetes, malignancy, coeliac disease, cystic fibrosis, no history of inflammation or acute infections requiring medical treatment or evaluation in the preceding six months, and no upper abdominal pain and nausea symptoms. Diagnosis of either acute pancreatitis or definite chronic pancreatitis was made based on international guidelines. Exclusion criteria were an attack of pancreatitis within three months before enrolment into this study, post-endoscopic retrograde cholangiopancreatography pancreatitis, hereditary pancreatitis, autoimmune pancreatitis, pancreatic cyst, congenital anomalies of the pancreas, cystic fibrosis, pancreatic lipomatosis or lipomatous pseudohypertrophy, malignancy, liver diseases, cognitive impairment, surgical, endoscopic, or radiological interventions involving the pancreas, steroid therapy, metallic body implants or other implantable electronic devices, or pregnancy. The acute pancreatitis group was made up of individuals after the index attack of acute pancreatitis. The chronic pancreatitis group consisted of individuals with definite or probable chronic pancreatitis, in line with the M-ANNHEIM classification and with a view to covering the entire disease spectrum of chronic pancreatitis [24]. All participants gave written informed consent.

### 2.2. Assessment of Iron Metabolism

Fasting (i.e., at least 8 h without food intake) venous blood samples were collected using EDTA tubes and centrifuged for 7.5 min to obtain plasma. Plasma hepcidin levels were measured using a solid-phase enzyme-linked immunosorbent assay (DRG^®^ Hepcidin 25 Bioactive, EIA-5258, DRG International, Inc., Springfield, NJ, USA) [25]. Plasma ferritin levels were measured using an electrochemiluminescence immunoassay (Roche^©^ Products and Roche Diagnostics Ltd., Basel, Switzerland). Habitual iron intake over the year prior to recruitment was assessed using the EPIC-Norfolk food frequency questionnaire [26]. The FETA software V.2.53 was used to calculate the intake of total, haem, and non-haem iron (mg/day) based on the frequency and portion sizes of 130 food items, as described elsewhere [27].

### 2.3. Imaging Protocol

**Figure 1:**
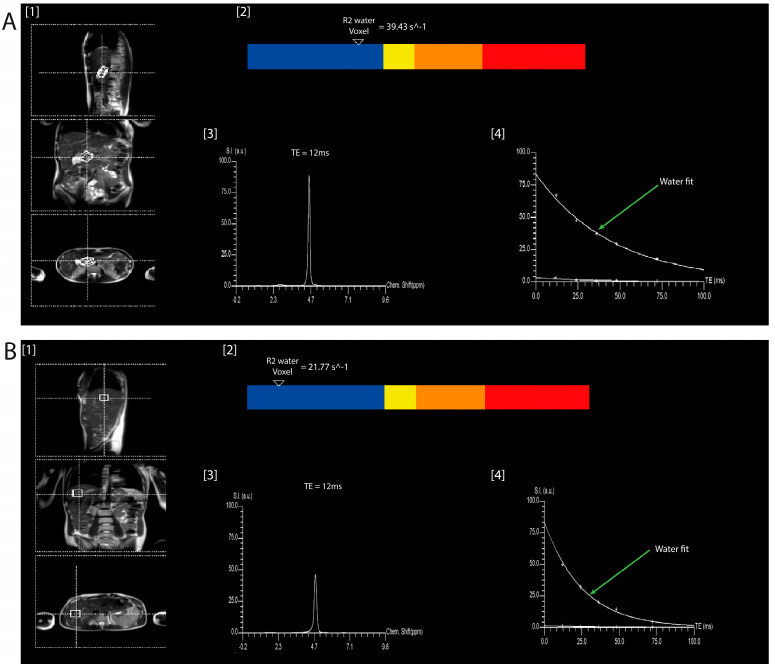
Determination of R2_water_ values with the use of T2-corrected multi-echo single-voxel (HISTO) sequence. Footnotes: The pancreas (**A**) and liver (**B**) R2_water_ measurements involved three principal steps. First, voxels were placed over the pancreas and liver (1). Second, results were displayed in colour bars, ranging from blue for the lowest value to red for the highest value of R2_water_ in the single voxel for the whole tissue volume (2). Third, the water spectra for TE = 12 ms (3) and graphs of T2 decay of water (4) were used to estimate the equilibrium signals.

Abdominal MR scans were performed on all participants using a 3.0 Tesla MAGNETOM Skyra^®^ scanner (Siemens, Erlangen, Germany). The selection of the MR spectroscopy region of interest was guided by the T1-weighted spin-echo MR images acquired in transverse and sagittal planes. High-speed T2-corrected multi-echo single-voxel (HISTO) ^1^H-MR spectroscopy sequence using a single voxel in a single breath hold was used with the echo times of 12, 24, 36, 48, and 72 ms. MR data were processed inline by using a spectrum with T2 correction. A report with quantitative information (i.e., pancreas R2_water_, liver R2_water_) displayed in both textual and colour bar formats was obtained (Figure 1). MR spectroscopy data were retrieved, exported as DICOM files, viewed using the MicroDicom DICOM Viewer software 2023.2 (32-bit) (MicroDicom Ltd., Sofia, Bulgaria), and recorded in a spreadsheet as pancreas and liver R2_water_ values.

### 2.4. Covariates

A standardised data collection form was used to collect information on age, sex, and body mass index (BMI) during the study visit. BMI (kg/m^2^) was calculated as weight in kilograms divided by the square of the height in metres. Glycated haemoglobin (mmol/mol) was measured immediately after blood collection on fresh and never frozen blood using the boronate affinity chromatography assay (Trinity Biotech, Wicklow, Ireland) [28].

### 2.5. Statistical Analysis

All statistical analyses were performed using SAS 9.4. (SAS Institute Inc., Cary, NC, USA). Continuous data were expressed as median and interquartile range (IQR), whereas categorical data were expressed as frequencies and percentages. Non-linear associations between variables were explored using generalised additive model analyses [29] separately in the three study groups (i.e., health, acute pancreatitis, and chronic pancreatitis). Three models were constructed for these analyses: Model 1 was unadjusted; Model 2 adjusted for age, sex, and BMI; and Model 3 adjusted for age, sex, BMI, and glycated haemoglobin. Three sets of associations were explored: the associations between tissue R2_water_ (pancreas and liver) and circulating markers of iron metabolism (ferritin and hepcidin); the associations between tissue R2_water_ (pancreas and liver) and dietary iron intake (total, haem, and non-haem iron); and the associations between circulating markers of iron metabolism (ferritin and hepcidin) and dietary iron intake (total, haem, and non-haem iron). Data were reported as the degrees of freedom with corresponding *p*-values. Statistical significance was deemed as *p*-values < 0.05.

## 3. Results

### 3.1. Characteristics of Participants

A total of 139 individuals were included in this study, of whom 47 belonged to the health group, 54—the acute pancreatitis group, and 38—the chronic pancreatitis group. Table 1 presents detailed characteristics of the study groups. Individuals with pancreatitis underwent MRI at a median of 16 months after their last hospitalisation for pancreatitis.

### 3.2. Associations of R2_water_ in the Health Group

The median (IQR) of pancreas R2_water_ and liver R2_water_ was 22.7 (21.3–24.1) s^−1^ and 36.9 (34.1–39.6) s^−1^, respectively. Pancreas R2_water_ had a significant association with liver R2_water_ in the most adjusted model (*p* < 0.001). Pancreas R2_water_ was significantly associated with ferritin in the most adjusted model (*p* < 0.001), whereas it was not significantly associated with hepcidin in any model. Liver R2_water_ was significantly associated with both ferritin (*p* = 0.02) and hepcidin (*p* < 0.01) in the most adjusted model. Both pancreas R2_water_ and liver R2_water_ were significantly associated with total, haem, and non-haem iron intake in the most adjusted model (*p* < 0.001 for all). The same associations in models 1 and 2 are presented in Table 2. The associations between the studied circulating markers of iron metabolism and dietary iron intake are presented in Table 3.

### 3.3. Associations of R2_water_ in the Acute Pancreatitis Group

The median (IQR) of pancreas R2_water_ and liver R2_water_ was 23.3 (21.6–24.7) s^−1^ and 38.8 (35.0–42.4) s^−1^, respectively. Pancreas R2_water_ had a significant association with liver R2_water_ in the most adjusted model (*p* < 0.001). Pancreas R2_water_ also had a significant association with both ferritin (*p* < 0.001) and hepcidin (*p* < 0.001) in the most adjusted model. Liver R2_water_ had a significant association with hepcidin (*p* < 0.001), but not ferritin, in the most adjusted model. Pancreas R2_water_ was significantly associated with total and non-haem iron intake (both *p* < 0.001), but not haem iron intake, in the most adjusted model. The associations between liver R2_water_ and total, haem, and non-haem iron intake were not significant in the most adjusted model. The same associations in models 1 and 2 are presented in Table 2. The associations between the studied circulating markers of iron metabolism and dietary iron intake are presented in Table 3.

### 3.4. Associations of R2_water_ in the Chronic Pancreatitis Group

The median (IQR) of pancreas R2_water_ and liver R2_water_ was 22.9 (21.5–23.6) s^−1^ and 38.1 (35.7–40.9) s^−1^, respectively. Pancreas R2_water_ had a significant association with liver R2_water_ in the most adjusted model (*p* < 0.001). Pancreas R2_water_ had significant associations with both ferritin (*p* = 0.01) and hepcidin (*p* = 0.04) in the most adjusted model. Similarly, liver R2_water_ was significantly associated with both ferritin (*p* = 0.040) and hepcidin (*p* < 0.001) in the most adjusted model. Pancreas R2_water_ was significantly associated with total iron intake in the most adjusted model (*p* < 0.001) but not with haem or non-haem iron intake in the most adjusted model. Liver R2_water_ was significantly associated with total (*p* < 0.001), haem (*p* = 0.02), and non-haem (*p* < 0.001) iron intake in the most adjusted model. The same associations in models 1 and 2 are presented in Table 2. The associations between the studied circulating markers of iron metabolism and dietary iron intake are presented in Table 3.

## 4. Discussion

**Figure 2:**
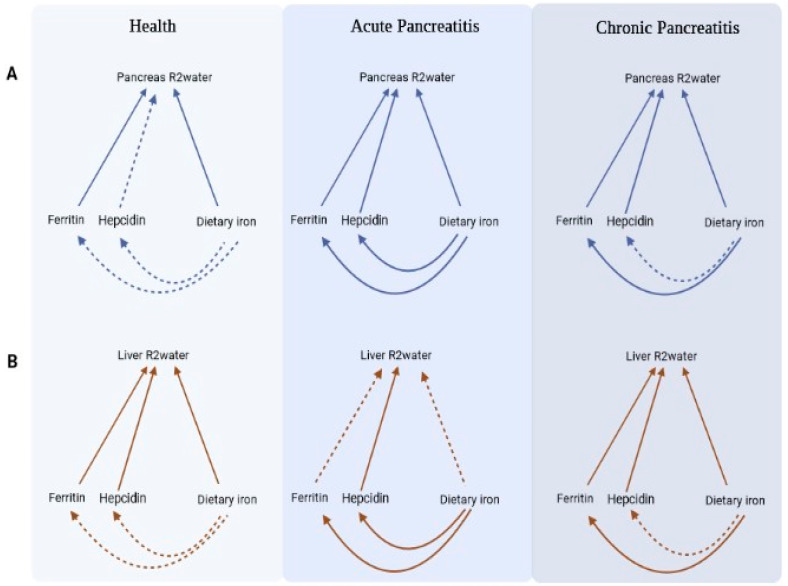
Summary of key findings in regard to pancreas R2_water_ (**A**) and liver R2_water_ (**B**). Footnotes: Solid arrows represent statistically significant (*p* < 0.05) associations, whereas dashed lines represent non-significant associations (after adjustment for age, sex, body mass index, and glycated haemoglobin). Dietary iron means total iron intake in this figure. All associations were from generalised additive model.

Iron is involved in one of the classical pathways of generating reactive oxygen species—the Fenton reaction [30,31]. For the first time, the present study investigated the associations between iron metabolism (assessed holistically through both dietary intake and circulating markers) and pancreas R2_water_ in health and pancreatitis—a common disease in which oxidative stress plays a role. The use of generalised additive model analyses adjusted for age, sex, BMI, and glycated haemoglobin enabled the exploration of non-linear associations between variables while taking into account possible confounders. One of the advantages of using a generalised additive model is its flexibility in regard to the assumption of linearity, allowing the model to adapt its fit based on the data [29]. Therefore, this statistical approach facilitates the identification of diverse patterns of association that could be otherwise overlooked if a linear regression model is employed. The other strength of the present study relates to the use of multi-echo MR spectroscopy (with T2 correction and 5 TEs fixed in the range from 12 to 72 ms) that separates the water resonance from confounding proton resonance such as lipids, enabling a separate display of water and lipids spectral peaks [16,18]. Further, the HISTO sequence facilitates a reproducible, quick, and automated measurement of highly resolved water spectra in the tissues [16]. One of the key findings in the present study was significant associations of pancreas R2_water_ with both total dietary intake of iron and circulating markers of iron metabolism (specifically, ferritin). This was observed consistently in both healthy individuals and individuals after acute pancreatitis and individuals after chronic pancreatitis (Figure 2). A significant association between pancreas R2_water_ and liver R2_water_ in all the study groups complemented these findings. Notably, all the above associations held true after accounting for possible confounders such as age, sex, BMI, and glycated haemoglobin.

The HISTO sequence has emerged as a robust and reproducible method for estimating R2 of the water component in tissue, which showed strong correlations with iron levels both in phantoms and in vivo [16,18,32]. This technique acquires spectral data at multiple echo times, enabling a T2 curve fit of water and lipids spectra and a subsequent T2 correction of the resultant water fraction quantity [18]. Multiple studies have shown a significant positive correlation between liver R2_water_ and liver iron concentrations [16,18,22]. Pineda et al. determined the transverse relaxation time for liver water (T2) using the HISTO in 25 people with high BMI and elevated liver enzymes. The authors found a strong significant linear association between liver R2_water_ and the amount of iron in the phantoms, which was independent of liver lipids [18]. Lin et al. determined liver R2_water_ in 38 individuals with hyperferritinaemia and verified the data using R2 and liver iron concentration from the commercially available FerriScan^®^ service. The authors found that liver R2_water_ and R2 derived from FerriScan^®^ had a strong positive correlation [32]. Sharma et al. used the HISTO in phantoms and 12 individuals. A significant linear relationship was found between liver R2_water_ and iron content in phantoms, with no dependence on lipid content [16]. Zhan et al. investigated the liver biopsies of 80 people who also had liver R2_water_ determined using the HISTO [22]. Liver R2_water_ had high accuracy in detecting iron overload [22]. In the present study, liver R2_water_ and pancreas R2_water_ were significantly associated, consistently in both health, acute pancreatitis, and chronic pancreatitis (after accounting for possible confounders). Although pancreas iron concentration per se was not investigated in the present study, the above indirect evidence suggests that pancreas R2_water_ may be a non-invasive marker of intra-pancreatic iron deposition. It is also reassuring that, in all the study groups, pancreas R2_water_ was significantly associated with ferritin (Figure 2)—the circulating marker of iron status most widely used in routine clinical practice.

Several associations of pancreas R2_water_ showed a differing pattern of relationship between the study groups. Specifically, pancreas R2_water_ was significantly associated with hepcidin in the acute and chronic pancreatitis groups but not in the health group. Hepcidin is the master hormone regulating iron balance by inhibiting iron uptake from the gut and iron recycling by macrophages, consequently decreasing iron levels in plasma [33,34,35]. Low levels of hepcidin lead to increased iron deposition within tissues, including the exocrine pancreas [36,37]. Dysfunctional iron metabolism has long been thought to play a part in pancreatitis, and the involvement of the exocrine pancreas in iron absorption and maintenance of systemic iron homeostasis has been confirmed in earlier human and animal studies [38,39,40]. More recent studies demonstrated increased hepcidin and decreased ferritin levels in individuals with glucose derangements after acute pancreatitis [39]. Further, histological studies of the pancreas showed a statistically significant increase in iron per gram of pancreatic tissue in individuals with chronic pancreatitis (without overt iron overload) compared with healthy individuals [41]. Another notable finding in the present study was the differences in the associations of pancreas R2_water_ with components of dietary iron intake (i.e., total, haem, and non-haem iron). In both the health and acute pancreatitis groups, non-haem iron intake was significantly associated with pancreas R2_water._ By contrast, this association was not statistically significant in the chronic pancreatitis group. Of note, non-haem iron intake was also significantly associated with hepcidin in the acute pancreatitis group but not the chronic pancreatitis group (Table 3). These observations could be explained by the differences in chemical structure, bioavailability, and absorptive properties between non-haem iron (inorganic, Fe^3+^) and haem iron (organic, Fe^+^). While non-haem iron is predominantly sourced from vegetables and cereals, haem iron is derived from animal sources such as red meat, chicken, and fish [42]. During digestion, the readily absorbable haem iron is taken up as a metalloporphyrin by intestinal cells through endocytosis, whereas non-haem iron must first be reduced to a ferrous form by duodenal reductases for absorption to occur [43,44]. Haem is maintained soluble for absorption by globin degradation products produced by pancreatic enzymes, which help to release haem from haemoglobin and myoglobin [45,46,47]. Therefore, the presence of exocrine pancreatic dysfunction in chronic pancreatitis may explain the disruptions of iron absorption in the duodenum that are associated with the progression of pancreatitis [39,48]. In addition, as pancreatic β-cells were reported to be an extra-hepatic source of hepcidin [49], the progression of pancreatitis may drive further the observed derangements of iron metabolism.

This study has several limitations. First, one could argue that pancreas R2* (which is calculated from GRE sequences) is a more promising imaging biomarker of iron metabolism. Unfortunately, it was not investigated in the present study as we used a spin echo sequence. However, given that an earlier liver study found a significant correlation between liver R2_water_ and R2* [32] and taking into account the finding of a significant association between liver R2_water_ and pancreas R2_water_ in the present study, it is conceivable that pancreas R2_water_ may serve as a complementary imaging marker potentially useful in the imaging work-up of the pancreas. Other emerging imaging markers include radiomics of the pancreas [50,51] and intra-pancreatic fat deposition [52]. Second, the HISTO sequence yielded tissue R2_water_ in a single voxel, which might have led to a sampling error if pancreas R2_water_ were not evenly distributed throughout the organ. However, given that a large 2022 study showed a homogenous distribution of fat within the pancreas [53], it is very likely that pancreas R2_water_ is also distributed evenly. Third, histological data were not available as pancreas biopsy would have been unethical (and possibly harmful) in this study’s population. The presented findings should be viewed as hypothesis-generating, ultimately paving the way to purposely-designed studies that investigate the associations between pancreas iron content and MR imaging markers. Fourth, the chronic pancreatitis group had a relatively small sample size and an imbalance in terms of sex. However, our sample size reflects the low prevalence of chronic pancreatitis in the general population [2]. It is also worth noting that all analyses in the present study were adjusted for sex to account for its possible confounding effect. Last, the cross-sectional nature of the present study precludes establishing causality. Longitudinal cohort studies are warranted to investigate temporal changes in pancreas R2_water_ with a view to drawing causal inferences.

In conclusion, the present study of more than one hundred individuals provided the first data on the relevance of MR-derived pancreas R2_water_ to iron metabolism. Further, the differing patterns of association between pancreas R2_water_ and non-haem iron intake, as well as circulating levels of hepcidin, suggest the possible role of changes in pancreas water content and iron metabolism in the progression of pancreatitis. Measurement of pancreas R2_water_ using the multi-echo HISTO technique could potentially be used in the clinic with a view to non-invasive assessment of intra-pancreatic iron deposition in the future.

## Figures and Tables

**Table 1:** Characteristics of this study’s groups.

Characteristic	Health (n = 47)	Acute Pancreatitis (n = 54)	Chronic Pancreatitis (n = 38)	*p*-Value
Age (years)	55 (32–69)	58 (46–64)	58 (49-66)	0.524
Sex				**0.002**
Men	19 (40%)	31 (57%)	30 (79%)	
Women	28 (60%)	23 (42.6%)	8 (21%)	
BMI (kg/m^2^)	24.2 (21.6–28.1)	27.5 (24.9–33.1)	28 (24–33)	**0.002**
HbA1c (mmol/mol)	36 (33.0–38.0)	39 (36–42)	41 (38–51)	**<0.001**
Ferritin (ng/mL)	114 (55–181)	130 (87–243)	192 (104–314.5)	0.050
Hepcidin (ng/mL)	11.3 (4.4–15.0)	10.1 (8.0–24.6)	14.0 (11.7–21.5)	0.089
Total iron intake (mg/day)	10.2 (8.2–15.1)	10.0 (7.1–12.6)	9.7 (7.4–15.2)	0.735
Haem iron intake (mg/day)	0.6 (0.4–1.0)	0.7 (0.6–1.2)	0.8 (0.5–1.1)	0.190
Non-haem iron intake (mg/day)	9.6 (7.8–14.2)	8.6 (6.6–11.7)	9.0 (6.6–13.9)	0.633

Footnotes: Data are presented as median and interquartile range or count frequency and percentage. Statistically significant associations (*p* < 0.05) are in bold. Abbreviations: BMI; body mass index; HbA1c; glycated haemoglobin.

**Table 2:** Associations of tissue R2_water_ with circulating markers of iron metabolism and dietary iron intake.

			Circulating Marker				Dietary Intake (mg/day)					
Tissue R2_water_	Group	Model	Ferritin		Hepcidin		Total Iron		Haem Iron		Non-Haem	
			DF	*p*-Value	DF	*p*-Value	DF	*p*-Value	DF	*p*-Value	DF	*p*-Value
Pancreas R2_water_												
	Health	Model 1	0.36	0.13	0.87	**0.04**	<0.001	**<0.001**	<0.001	**<0.001**	<0.001	**<0.001**
		Model 2	<0.001	**<0.001**	0.67	0.10	<0.001	**<0.001**	<0.001	**<0.001**	<0.001	**<0.001**
		Model 3	<0.001	**<0.001**	0.62	0.12	<0.001	**<0.001**	<0.001	**<0.001**	<0.001	**<0.001**
												
	AP	Model 1	<0.001	**<0.001**	<0.001	**<0.001**	<0.001	**<0.001**	0.06	0.07	<0.001	**<0.001**
		Model 2	<0.001	**<0.001**	<0.001	**<0.001**	<0.001	**<0.001**	0.54	0.12	<0.001	**<0.001**
		Model 3	<0.001	**<0.001**	<0.001	**<0.001**	<0.001	**<0.001**	0.58	0.12	<0.001	**<0.001**
												
	CP	Model 1	2.00	**<0.001**	2.00	**0.03**	<0.001	**<0.001**	0.01	**<0.001**	0.72	0.08
		Model 2	2.00	**0.01**	2.00	0.07	<0.001	**<0.001**	0.86	0.12	0.63	0.11
		Model 3	2.00	**0.01**	2.00	**0.04**	<0.001	**<0.001**	0.90	0.13	0.65	0.11
Liver R2_water_												
	Health	Model 1	5.33	**0.01**	<0.001	**<0.001**	<0.001	**<0.001**	<0.001	**<0.001**	<0.001	**<0.001**
		Model 2	5.32	**0.02**	<0.001	**<0.001**	<0.001	**<0.001**	<0.001	**<0.001**	<0.001	**<0.001**
		Model 3	5.15	**0.02**	<0.001	**<0.001**	<0.001	**<0.001**	<0.001	**<0.001**	<0.001	**<0.001**
												
	AP	Model 1	0.87	0.05	0.17	0.11	0.40	0.12	0.62	0.09	0.29	0.12
		Model 2	0.80	0.06	<0.001	**<0.001**	0.40	0.14	0.69	0.09	0.25	0.13
		Model 3	0.84	0.05	<0.001	**<0.001**	0.51	0.13	0.76	0.09	0.37	0.14
												
	CP	Model 1	1.35	**0.01**	<0.001	**<0.001**	<0.001	**<0.001**	0.98	**0.03**	<0.001	**<0.001**
		Model 2	1.42	**0.03**	<0.001	**<0.001**	<0.001	**<0.001**	0.97	0.05	<0.001	**<0.001**
		Model 3	1.43	**0.04**	<0.001	**<0.001**	<0.001	**<0.001**	1.22	**0.02**	<0.001	**<0.001**

Footnotes: Data are presented as degrees of freedom and *p*-values (from generalised additive model). Model 1: unadjusted. Model 2: adjusted for age, sex, and body mass index. Model 3: adjusted for age, sex, body mass index, and glycated haemoglobin. Statistically significant associations (*p* < 0.05) are in bold. Abbreviations: AP, acute pancreatitis; CP, chronic pancreatitis; DF, degrees of freedom.

**Table 3:** Associations of circulating markers of iron metabolism with dietary iron intake.

Circulating Marker		Model	Total Iron		Haem Iron		Non-Haem Iron	
	Group		DF	*p*-Value	DF	*p*-Value	DF	*p*-Value
Ferritin								
	Health	Model 1	<0.001	**<0.001**	0.46	0.10	<0.001	**<0.001**
		Model 2	0.89	0.09	0.61	0.10	0.85	0.10
		Model 3	0.68	0.11	0.61	0.11	0.70	0.11
	AP	Model 1	<0.001	**<0.001**	<0.001	**<0.001**	<0.001	**<0.001**
		Model 2	<0.001	**<0.001**	<0.001	**<0.001**	<0.001	**<0.001**
		Model 3	<0.001	**<0.001**	0.07	0.08	<0.001	**<0.001**
								
	CP	Model 1	<0.001	**<0.001**	<0.001	**<0.001**	<0.001	**<0.001**
		Model 2	<0.001	**<0.001**	<0.001	**<0.001**	<0.001	**<0.001**
		Model 3	<0.001	**<0.001**	<0.001	**<0.001**	<0.001	**<0.001**
Hepcidin								
	Health	Model 1	1.99	0.08	<0.001	**<0.001**	2.02	0.05
		Model 2	2.02	0.09	2.61	0.05	2.02	0.09
		Model 3	1.98	0.13	2.56	0.09	1.96	0.11
	AP	Model 1	<0.001	**<0.001**	<0.001	**<0.001**	<0.001	**<0.001**
		Model 2	<0.001	**<0.001**	<0.001	**<0.001**	<0.001	**<0.001**
		Model 3	<0.001	**<0.001**	<0.001	**<0.001**	<0.001	**<0.001**
	CP	Model 1	0.46	0.11	<0.001	**<0.001**	0.51	0.11
		Model 2	0.71	0.09	<0.001	**<0.001**	0.71	0.09
		Model 3	0.61	0.12	<0.001	**<0.001**	0.62	0.13

Footnotes: Data are presented as degrees of freedom and *p*-values (from generalised additive model). Model 1: unadjusted. Model 2: adjusted for age, sex, and body mass index. Model 3: adjusted for age, sex, body mass index, and glycated haemoglobin. Statistically significant associations (*p* < 0.05) are in bold. Abbreviations: AP, acute pancreatitis; CP, chronic pancreatitis; DF, degrees of freedom.

## Data Availability

The data are not publicly available due to the ethical regulations governing human research.
